# Supplementary material for: D-DOPA Is a Potent, Orally Bioavailable, Allosteric Inhibitor of Glutamate Carboxypeptidase II
Source: Pharmaceutics. 2022 Sep 23;14(10):2018. doi: 10.3390/pharmaceutics14102018 (PMC9608075; doi:10.3390/pharmaceutics14102018)
Supplement: Supplementary file 1 [file pharmaceutics-14-02018-s001.zip › pharmaceutics-1890935-supplementary.pdf]

Supporting Information

D-DOPA Is a Potent, Orally Bioavailable, Allosteric Inhibitor of Glutamate Carboxypeptidase II

Sadakatali S. Gori [1,2], Ajit G. Thomas [2], Arindom Pal [1,2], Robyn Wiseman [2,3], Dana V. Ferraris [2], Run-duo Gao [1,2], Ying Wu [2], Jesse Alt [2], Takashi Tsukamoto [1,2,3], Barbara S. Slusher [1,2,3,4,5,6,7,*] and Rana Rais [1,2,3,*]

1 Department of Neurology, Johns Hopkins School of Medicine, Baltimore, MD 21205, USA
2 Johns Hopkins Drug Discovery, Johns Hopkins School of Medicine, Baltimore, MD 21205, USA
3 Department of Pharmacology and Molecular Sciences, Johns Hopkins School of Medicine, Baltimore, MD 21205, USA
4 Departments of Psychiatry and Behavioral Sciences, Johns Hopkins School of Medicine, Baltimore, MD 21205, USA
5 Department of Oncology, Johns Hopkins School of Medicine, Baltimore, MD 21205, USA
6 Department of Neuroscience, Johns Hopkins School of Medicine, Baltimore, MD 21205, USA
7 Department of Medicine, Johns Hopkins School of Medicine, Baltimore, MD 21205, USA
* Correspondence: bslusher@jhmi.edu (B.S.S.); rrais2@jhmi.edu (R.R.)

Table of Contents

1. Metabolite Identification in D-/L-DOPA Spiked Brain Homogenate – Page 3
2. Suitability of LC-MS Method Used for D-DOPA Analyses – Page 4

<u>**Metabolite Identification in D-/L-DOPA Spiked Brain Homogenate**</u>

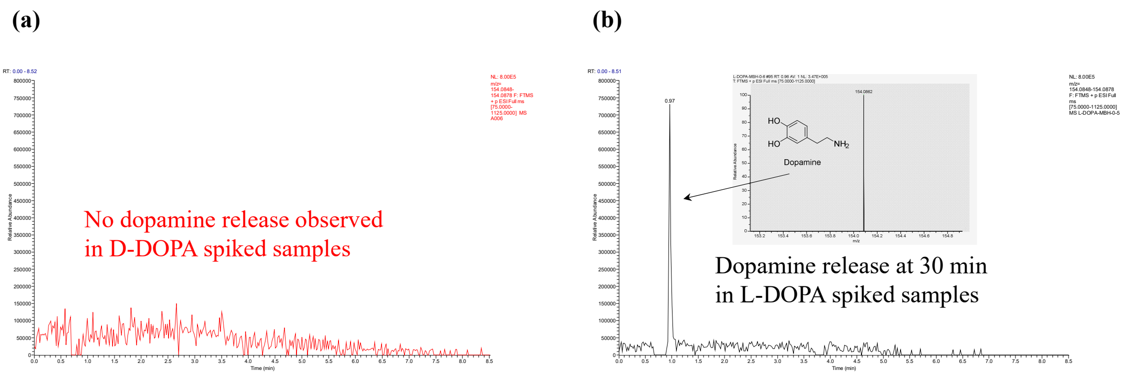

Figure S1. Dopamine release from (a) D-DOPA (red plot) and **(b)** L-DOPA (black plot) spiked brain homogenate at 30 min post-spike. Dopamine is observed in L-DOPA spiked brain homogenate and is not observed in D-DOPA spiked brain homogenate.

<u>Accuracy and Precision of LC-MS Method Used for D-DOPA Analyses in Plasma</u>

Table S1-A. Statistical evaluation of calculated concentrations of derivatized D-DOPA obtained from the calibration curves (n=3) prepared in mouse plasma and brain.

Nominal Concentration (nmol/mL)	Plasma		Brain	
	Accuracy (%)	Precision (% RSD)	Accuracy (%)	Precision (% RSD)
0.03	112	1.11	-	-
0.1	97.1	5.05	115	0.848
0.3	93.7	2.40	95.8	0.750
1	96.5	1.05	93.9	1.38
3	100	0.680	93.6	0.776
10	100	0.966	101	1.14
30	97.4	3.89	101	1.10
100	98.8	2.58	99.9	3.34

Table S1-B. Inter and intra-day precision and accuracy for derivatized D-DOPA in mouse plasma and brain determined by analyzing replicates (n=3/day) of spiked samples at 4 different concentration levels over 2 subsequent days. Statistics for inter-day evaluation are generated from n=6 samples.

Nominal Concentration (nmol/mL)	Plasma				Brain			
	Intraday (n=3)		Interday (n=6)		Intraday (n=3)		Interday (n=6)	
	Accuracy (%)	Precision (% RSD)	Accuracy (%)	Precision (% RSD)	Accuracy (%)	Precision (% RSD)	Accuracy (%)	Precision (% RSD)
0.05	108	0.831	108	0.831	-	-	-	-
0.5	94.0	2.75	94.0	2.75	95.5	2.48	95.0	2.14
5	100	3.88	100	3.88	95.5	1.82	97.4	2.52
50	101	6.09	101	6.09	101	0.586	101	1.06

Table S1-C. Stability of derivatized D-DOPA in mouse plasma and brain when stored at benchtop (18 h, room temperature) and autosampler (18 h at 4ºC).

Nominal Concentration (nmol/mL)	Plasma		Brain	
	Benchtop Stability (%)	Autosampler Stability (%)	Benchtop Stability (%)	Autosampler Stability (%)
0.05	101 ± 0.588	96.0 ± 3.17	-	-
0.5	101 ± 1.03	103 ± 1.59	97.7 ± 0.0152	98.4 ± 0.0115
5	100 ± 1.54	101 ± 2.22	102 ± 0.00415	102 ± 0.00557
50	98.9 ± 1.30	102 ± 3.17	99.6 ± 0.0126	99.4 ± 0.00743
